# MicroRNA-205 mediates endothelial progenitor functions in distraction osteogenesis by targeting the transcription regulator NOTCH2

**DOI:** 10.1186/s13287-021-02150-x

**Published:** 2021-02-03

**Authors:** Weidong Jiang, Peiqi Zhu, Tao Zhang, Fengchun Liao, Yangyang Yu, Yan Liu, Huijuan Shen, Zhenchen Zhao, Xuanping Huang, Nuo Zhou

**Affiliations:** 1 Guangxi Medical University, Nanning, 530021 People’s Republic of China; 2 Department of Oral and Maxillofacial Surgery, Hospital of Stomatology, Guangxi Medical University, Nanning, 530021 People’s Republic of China; 3 Guangxi Key Laboratory of Oral and Maxillofacial Rehabilitation and Reconstruction, Guangxi Key Laboratory of Oral and Maxillofacial Surgery Disease Treatment, Guangxi Clinical Research Center for Craniofacial Deformity, Nanning, 530021 People’s Republic of China

**Keywords:** Distraction osteogenesis, microRNA-205, NOTCH2, Endothelial colony-forming cells, Bone regeneration, Angiogenesis

## Abstract

**Background:**

Distraction osteogenesis (DO) is a highly efficacious form of reconstructive bone regeneration, but its clinical utility is limited by the prolonged period required for bone consolidation to occur. Understanding the mechanistic basis for DO and shortening this consolidation phase thus represent promising approaches to improving the clinical utility of this procedure.

**Methods:**

A mandibular DO (MDO) canine model was established, after which small RNA sequencing was performed to identify relevant molecular targets genes. Putative miRNA target genes were identified through bioinformatics and confirmed through qPCR, Western blotting, and dual-luciferase reporter assays. Peripheral blood samples were collected to isolate serum and endothelial colony-forming cells (ECFCs) in order to measure miR-205, NOTCH2, and angiogenic cytokines expression levels. Lentiviral constructs were then used to inhibit or overexpress miR-205 and NOTCH2 in isolated ECFCs, after which the angiogenic activity of these cells was evaluated in migration, wound healing, proliferation, tube formation, and chick chorioallantoic membrane (CAM) assay. Autologous ECFCs transfected to knockdown miR-205 and were injected directly into the distraction callus. On days 14, 28, 35 and 42 after surgery, bone density was evaluated via CBCT, and callus samples were collected and evaluated via histological staining to analyze bone regeneration and remodeling.

**Results:**

MiR-205 was identified as being one of the miRNAs that was most significantly downregulated in MDO callus samples. Downregulation of miR-205 was also observed in DO-ECFCs and serum of animals undergoing MDO. Inhibiting miR-205 markedly enhanced angiogenesis, whereas overexpressing miR-205 had the opposite effect in vitro*.* Importantly, NOTCH2, which is a unique regulator in bone angiogenesis, was identified as a miR-205 target gene. Consistent with this regulatory relationship, knocking down NOTCH2 suppressed angiogenesis, and transduction with a miR-205 inhibitor lentivirus was sufficient to rescue angiogenic activity. When ECFCs in which miR-205 had been inhibited were transplanted into the MDO callus, this significantly bolstered osteogenesis, and remodeling in vivo.

**Conclusions:**

MiR-205 is a significant regulator of the MDO process, and inhibiting this miRNA can accelerate MDO-related mineralization. Overall, these results offer new insights into the mechanistic basis for this procedure, highlighting potential targets for therapeutic clinical intervention.

**Supplementary Information:**

The online version contains supplementary material available at 10.1186/s13287-021-02150-x.

## Background

Distraction osteogenesis (DO) is an effective approach to the treatment of bone defects, orthopedic disorders, and craniomaxillofacial deformities [1, 2]. DO consists of the gradual distraction of osteotomy cuts, resulting in the formation of new bone between these gaps. While efficacious, DO is a slow process that requires a prolonged consolidation phase, and it is also associated with increased complication risks. Clinical approaches to shortening the consolidation phase are thus of clear value, and it is therefore vital that the mechanistic basis for DO be better clarified.

Bone regeneration is highly dependent upon the angiogenic remodeling of extant vascular structures and on vasculogenesis, wherein new blood vessels are formed [3]. During consolidation, inadequate blood supply is a major cause of delayed bone union and non-union, leading to many of the complications associated with this process [4]. Endothelial progenitor cells (EPCs) undergo differentiation to yield the endothelial cells necessary for vasculogenesis to occur. In the context of tissue repair and remodeling, EPCs home to injured vascular sites and support ongoing angiogenesis [5]. In engineered bone tissue, EPCs can support neovascularization, thereby enhancing osteogenesis and bone reconstruction [6, 7]. There is also evidence that EPCs are important mediators of DO and fracture healing, given that enhanced EPC mobilization can accelerate osteogenesis in vitro [8] and can promote neovascularization and new bone formation [9, 10]. Endothelial colony-forming cells (ECFCs), which are also known as late EPCs, have characteristics of true endothelial progenitor cells [11, 12], developing vascular networks and integrating into the nascent vasculature in vivo*.* Accelerating bone regeneration by studying angiogenesis hold great promise, but the underlying mechanisms remain unclear, highlighting further need for study of this issue.

MicroRNAs (miRNAs) are small (18–22 nucleotide) RNAs which lack coding potential but can regulate the expression of their complementary target mRNAs in a sequence-specific manner [13]. Specific miRNAs have been shown to control the balance been pro- and anti-angiogenic activity in a range of contexts [14], and there is also experimental evidence suggesting the importance of miRNAs as regulators of angiogenic-osteogenic coupling [15]. For example, miR-9 [16], miR-26a [17], and miR-210 [18] have all been shown to be drivers of angiogenesis and osteogenesis, while miR-10a [19, 20], miR-200b [21], and miR-497-195 cluster members [22] can suppress these same processes. As such, specific miRNAs likely control angiogenesis in the context of DO and may represent potent therapeutic targets that can be leveraged to promote bone development. In this study, we have identified multiple differentially regulated miRNAs in a mandibular DO (MDO) model system, with miR-205 being particularly downregulated in the context of MDO. Prior work suggests that inhibiting miR-205 can promote bone mesenchymal stem cell (BMSC) osteogenic differentiation through the regulation of the SATB2/Runx2 and ERK/MAPK pathways [23]. The role of miR-205 in oncogenic contexts is more complex, as it can function as a tumor suppressor that inhibits angiogenesis and proliferation in certain contexts [24], while in others it can promote both tumorigenesis and angiogenesis [25]. Therefore, miR-205 may represent a potent and clinically relevant regulator of angiogenesis in the context of DO. However, whether miR-205 regulates the angiogenic activity of ECFCs in the context of DO remains to be established.

The only miRNAs to have been studied as regulators of DO to date are miR-503 [26] and miR-144-3p [27], which have been shown to function as regulators of bone development in limb DO. Herein, we explored the potential role of miR-205 as a novel regulator of neovascularization during MDO. Using a canine MDO model system, we found that miR-205 inhibition was sufficient to enhance angiogenesis in the context of mandibular regeneration in vivo. Inhibiting this miRNA in ECFCs in vitro was also sufficient to elevate their migration, proliferation, and tube forming activity. Importantly, we identified NOTCH2 as a novel miR-205 target gene that was directly associated with DO-related angiogenic activity. Overall, our data highlight miR-205 as a promising therapeutic target that may be used to develop new approaches to clinical bone regeneration.

## Methods

### Animals

This study was conducted using 1-year-old male Beagle dogs (*n* = 5 for ECFC harvesting; *n* = 29 for MDO modeling) from the Experimental Animal Center of Guangxi Medical University (Nanning, China). The Animal Care and Use Committee of Guangxi Medical University approved this study. All animals were housed in iron cases in a climate-controlled environment (25 ± 3 °C, constant humidity) with free access to standard chow and sterile water.

### MDO model establishment

This canine MDO model was established as in prior studies [10, 28]. Briefly, dogs were anesthetized via the intraperitoneal injection of pentobarbital (1 mg/kg) and xylazine (2 mg/kg). The operative site was then shaved, sterilized using 0.5% iodine, and injected with a solution containing 0.5% lidocaine and 1:200,000 epinephrine. A 5-cm incision was then made on the right side from the midline on the inferior border of the mandible through the skin, and dissection through subcutaneous and muscular layers was performed. Care was taken to avoid damaging the facial artery. Careful dissection of the periosteum was then performed to expose the lateral aspect of the mandibular body (Supplemental Fig. S1A, a). An osteotomy line was drawn on the mandibular first and second molar (Supplemental Fig. S1A, b). The distraction device was then installed such that the original mandibular position could be assessed following osteotomy (Supplemental Fig. S1A, c). During osteotomy, care was taken to avoid damaging the inferior alveolar neurovascular bundle. After osteotomy was complete, the distal and proximal segments were fixed with an internal distraction fixator (Cibei, China) (Supplemental Fig. S1A, d, e). Lastly, 4/0 polyglactin absorbable sutures were used to close the mandibular skin (Supplemental Fig. S1A, f). Dogs were intramuscularly administered cephalosporin and tramadol hydrochloride for 3 days after surgery. Beginning 7 days post-surgery, distraction was performed at a 1-mm/day rate in two 12-h steps for 7 days. On day 14 post-surgery, 9 dogs were euthanized for analysis, while all other animals were assessed on days 28 and 42 postoperatively.

### RNA sequencing and PCR

Callus tissue sample RNA was extracted using TRIzol (Invitrogen, CA, USA) based on provided directions, after which a small RNA (sRNA) sequencing library was prepared based on instructions provided with the Illumina Small RNA Prep kit. Small RNA-seq was performed at the Illumina Hiseq 2000 instrument (Illumina Inc., CA). All cDNA synthesis was conducted with a reverse transcription kit (Invitrogen) for mRNAs and a Mir-X™ miRNA First-Strand Synthesis kit (Takara, Japan) for miRNAs. The expression of these RNA molecules was evaluated via qPCR (QuantStudio-5 system, Applied Biosystems) with the 2×PowerUp SYBR Green Master Mix (Invitrogen) using primers shown in Table 1. U6 and β-actin served as normalization controls for miRNA and mRNA expression, respectively, with the 2^−ΔΔCt^ method being used to quantify relative gene expression.

**Table 1:** Primers sequences used in the RT-qPCR

Gene	Sequences
MiR-141	5′-CCGCAACACTGTCTGGTAAAGATGG-3′
MiR-205	5′-CTTCATTCCACCGGAGTCTGA-3′
MiR-196b	5′-CGCTAGGTAGTTTCCTGTTGTTGGGA-3′
MiR-33a	5′-CGCGTGCATTGTAGTTGCATTGC-3′
MiR-206	5′-CGCTGGAATGTAAGGAAGTGTGTGG-3′
MiR-133c	5′-TTGGTCCCCTTCAACCAGCTG-3′
MiR-147	5′-GGTGTGCGGAAATGCTTCTGCTA-3′
MiR-503	5′-CGTAGCAGCGGGAACAGTACTG-3′
MiR-153	5′-CGGCGTTGCATAGTCACAAAAGTGA-3′
MiR-219-5p	5′-CGCCTGATTGTCCAAACGCAATTCT-3′
VEGF-A-F	5′-TCCACCATGCCAAGTGGT-3′
VEGF-A-R	5′-CCATGAACTTCACCACTTCG-3′
bFGF-F	5′-AGAGAGCGTTGTGTCCATC-3′
bFGF-R	5′-GCCCAGTTCGTTTCAGTGC-3′
NOTCH2-F	5′-TGGGCAGCTGCTGTCAATAA-3′
NOTCH2-R	5′-ATGAGGAGCACCCCTCACTTT-3′
U6-F	5′-GGAACGATACAGAGAAGATTAGC-3′
U6-R	5′-TGGAACGCTTCACGAATTTGCG-3′
β-actin-F	5′-GCAAGGACCTCTATGCCAACA-3′
β-actin-R	5′-GAAGCATTTGCGGTGGACG-3′

*F* forward, *R* reverse

### ECFC isolation and culture

Canine peripheral blood samples (40 mL) were used to isolate mononuclear cells (MNCs) via canine peripheral blood mononuclear cell isolation kit (Solarbio, China) density gradient centrifugation, after which MNCs were cultured on fibronectin-coated T25 cell dishes containing EGM2 medium (EGM-2; Lonza, USA) at 37 °C in a 5%CO_2_ humidified incubator. The phenotypes of ECFC were examined via flow cytometry using antibodies against mouse CD31, CD34, CD45 CD105, and CD133 (Invitrogen, USA). Dil-ac-LDL uptake and FITC-UEA-1 binding assays were conducted to further investigate the characteristics of ECFC.

### Lentiviral transduction

ECFCs were grown for 3 days, after which they were transduced with an appropriate lentiviral vector (1 × 10^8^ TU/mL) transfection. A 50 MOI value was used to identify optimal doses for the cfa-mir-205 inhibition, overexpression (OE), and NOTCH2 inhibition lentiviral vectors (GeneChem Corporation, Shanghai, China).

### Dual-luciferase reporter assay

Canine NOTCH2 3′-UTR WT of mutant PCR products were cloned into the pmirGLO vector (Promega, USA) to yield pmirGLO-NOTCH2-WT and pmirGLO-NOTCH2-MUT plasmids, which were then transfected into HEK293T cells along with miR-205 overexpression or control vectors. Cells were then incubated for 48 h at 37 °C, after which luciferase activity was assessed with a dual-luciferase reporter assay system (Promega) based on provided directions.

### Tube formation assay

Matrigel (Corning Co. Ltd., USA) was dissolved at 4 °C, added to a μ-Slide Angiogenesis plate (10 μL/well), and was then solidified via a 30 min incubation at 37 °C in a standard tissue culture incubator. Appropriately treated ECFCs were then added to this plate (1.5 × 10^4^ cells/well) and incubated for 6 h, after which tube formation was assessed via light microscope (× 100) (Nikon, Japan), and ImageJ was used to quantify the numbers of branches corresponding to the degree of in vitro angiogenesis.

### Transwell assay

Transwell assay inserts (Corning, NY, USA) were used to assess cellular migration. Briefly, ECFCs (8 × 10^3^ cells in 400 μL serum-free media) were added to the upper portion of these inserts in a 24-well plate, with 700 μL of complete media in the lower chamber. Cells were cultured for 24 h, after which 4% neutral-buffered formalin was used to fix all cells that had migrated to the lower chamber, which were subsequently stained using 0.1% crystal violet (Solarbio). Cells were then imaged via microscopy and counted.

### Wound healing assay

Cells were plated in 6-well plates (1.5 × 10^5^ cells/well) and grown to 80% confluence, at which time media was removed and cells were treated overnight with serum-free media. A sterile micropipette tip was then used to generate a straight scratch wound in the monolayer surface. Damaged cells were washed away using PBS, and serum-free media was then added. Cells were imaged after 0 and 24 h via microscopy, and ImageJ was used to quantify cellular migration.

### Cell counting kit-8 assay

Cell proliferation was monitored using a CCK-8 kit (Dojindo, Japan) based on provided directions. ECFCs were plated in 96-well plates (3 × 10^3^ cells/well in 100 μl of EGM), after which CCK-8 reagent was added at appropriate time points. Plates were then incubated for 3 h at 37 °C, after which a spectrophotometer (Infinite M Flex, TECAN) was used to quantify absorbance at 450 nm.

### Chick chorioallantoic membrane (CAM) assay

After being cultured at 37 °C for 7 days, a window was opened on the shell of fertilized eggs to expose the CAM and treated ECFCs were then added into the CAM. Next, tape was used to cover the window, and eggs were incubated at 37 °C under 60% humidity. Three days later, the CAM was fixed in 3.7% formaldehyde, and we visualized the results under a stereomicroscope (SMZ745T, Nikon, Japan). The vessel density was quantified by AngioTool.

### Western blotting

Cells were lysed on day 3 post-transfection using RIPA buffer containing protease inhibitors. Lysates were spun at 12,000×*g* for 15 min at 4 °C, after which supernatants were evaluated via BCA assay (Beyotime) to measure protein levels therein. Protein samples were then separated via SDS-PAGE and transferred to PVDF membranes (Millipore, MA, USA) that were blocked using 5% non-fat milk in TBST for 1 h at room temperature. Blots were then probed with anti-VEGF-A (Invitrogen, Cat. MA1–16629), anti-NOTCH2 (Beyotime, Cat. AF7590), or anti-FGF (AVIVA SYSTEMS BIOLOGY, Cat. ARP42005_P050) overnight at 4 °C, after which they were incubated for 2 h on the following day with appropriate secondary antibodies. An ECL reagent (Beyotime) was then used to detect protein bands for these samples.

### Enzyme-linked immunosorbent assay (ELISA)

VEGF protein levels in ECFC culture media and serum samples were quantified with a canine VEGF ELISA kit (Jianglai Bio, China) based on provided directions.

### Histology and immunohistochemistry analysis

The DO callus tissue samples from each group were decalcified for 4–8 weeks using 10% EDTA, after which they were paraffin-embedded and cut into 4-μm sections with a rotary microtome (RM2255, Leica, Germany). These sections were then deparaffinized using xylene, hydrated using an ethanol gradient, and subjected to H&E and immunohistochemical staining to evaluate the ability of regeneration with an inverted microscope. For immunohistochemical staining, mouse anti-VEGF (Invitrogen, 1:50, Cat. MA1-16629) and rabbit anti-CD31 (Bioss, 1:100, Cat. bs-0195R) were used to detect the expression of vascular markers.

### CBCT scans

CBCT scans (KAVO, USA) were conducted for dogs on days 14, 28, 35, and 42 of the DO processes using consistent exposure parameters and machine settings. iCATVision software was used to convert all data into a digital format.

### Statistical analysis

All experiments were conducted in triplicate. Data are given as means with standard deviations and were compared using unpaired Student’s *t* tests or repeated measured ANOVAs as appropriate, with *P* < 0.05 as the threshold of significance.

## Results

### MiR-205 expression levels are reduced during distraction osteogenesis

**Fig. 1:**
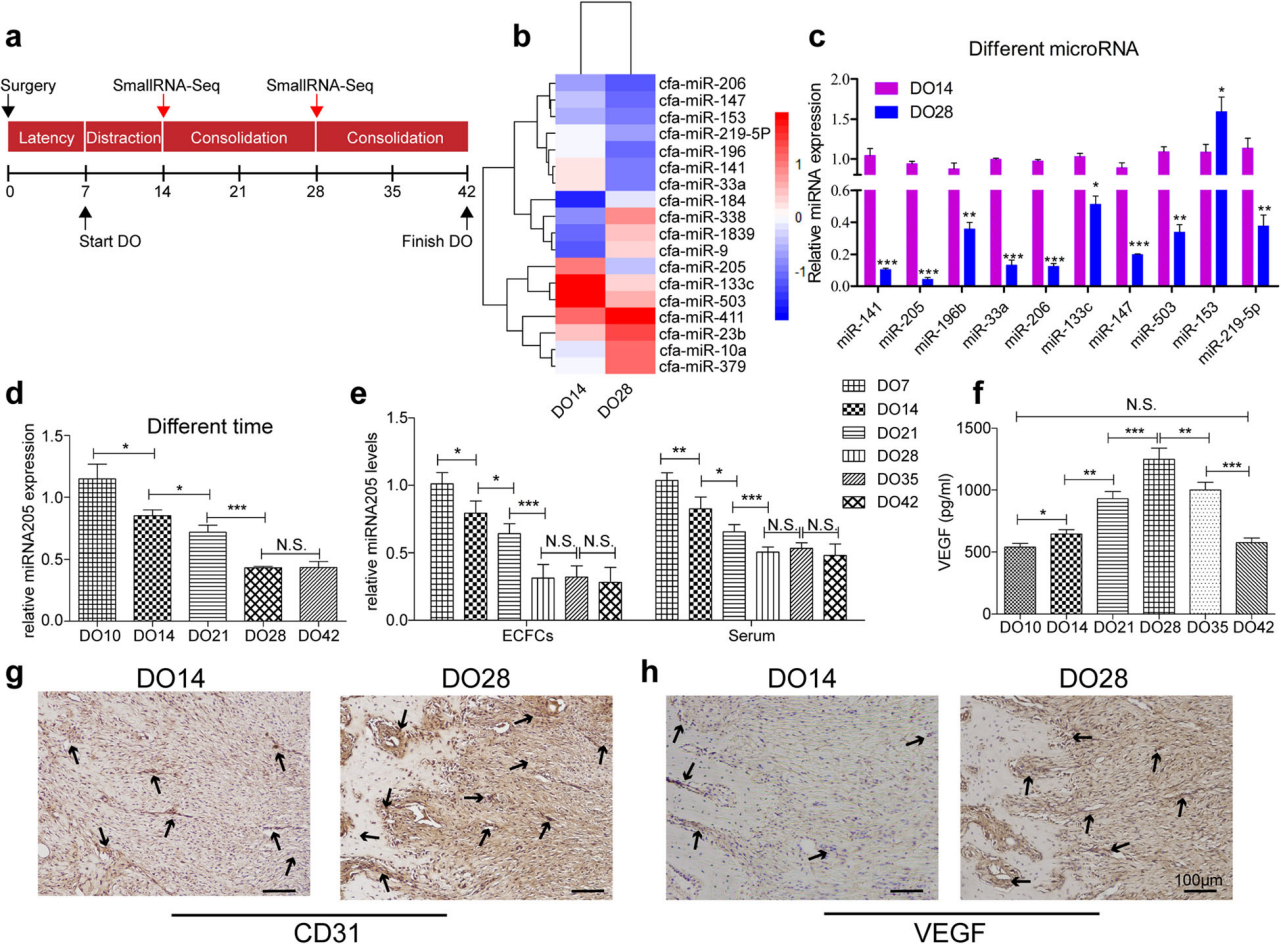
MiR-205 expression is decreased during the process of DO. **a** Canine model of MDO. At the end of the distraction phase (DO14) and 2 weeks of consolidation (DO28), callus samples from the distraction gap were harvested for small RNA-seq. **b** Heatmap of small RNA-seq results. The top ten up- or downregulated miRNAs were identified. **c** qPCR results revealed that miR-205 exhibited the greatest fold-change in expression levels (*n* = 10 per group). **d** The expression of miR-205 during different stages of DO. The expression of miR-205 gradually decreased before finally plateauing after 4 weeks of consolidation (day 42). **e** ECFCs and serum isolated on DO28 were found to express less miR-205 than those from DO21, DO14, and DO7 samples, with the expression of this miRNA decreasing over time. **f** The levels of circulating VEGF in serum from DO28 animals were also significantly higher than in serum from other DO time points. **g**, **h** Vascular markers (CD31 and VEGF) were upregulated in the distraction callus during DO (N.S, no significance; *P < 0.05, **P < 0.01 and ***P < 0.001)

We began by establishing a canine MDO model (Fig. 1a) in order to identify miRNAs controlling gene expression in the context of DO. We then performed small RNA-seq on days 14 and 28 at the end of the distraction and consolidation phases, respectively. This analysis revealed that the top 20 differentially expressed miRNAs were substantially differentially expressed on day 14 relative to day 28 (Fig. 1b). When miR-205 expression levels were assessed in study animals at different time points, it was found to be the most highly downregulated miRNA from a fold-change perspective on days 14 and 28 (Fig. 1c). The expression of miR-205 continued to gradually decrease over time, plateauing following 4 weeks of consolidation on day 42 (Fig. 1d). One prior study demonstrated that endothelial progenitor cells home to the DO site in large numbers during both the activation and consolidation phases [29]. As such, we next explored the potential role of miR-205 in DO angiogenesis by assessing its expression in ECFCs and serum samples collected from animals at different time points during the DO process. ECFCs isolated on DO day 28 (DO28) exhibited significantly reduced miR-205 expression relative to samples isolated on DO7, DO14, and DO21, with the expression of this miRNA having steadily declined over time. Serum miR-205 levels were also significantly lower on DO-28 relative to other analyzed time points (Fig. 1e). In contrast, serum VEGF levels rose gradually from DO14 to DO28, peaking on DO28 before declining to baseline on DO4442 (Fig. 1f). Increased expression of vascular marker proteins (VEGF and CD31) was also observed in the distraction callus over time (Fig. 1g, h). Together, these results suggested that ECFC angiogenesis are associated with bone development in the context of DO. In addition, the observed downregulation of miR-205 during DO may suggest that this miRNA regulates ECFC angiogenesis and associated osteogenic activity.

### MiR205 mediate ECFC tube formation, migration and proliferation in vitro

**Fig. 2:**
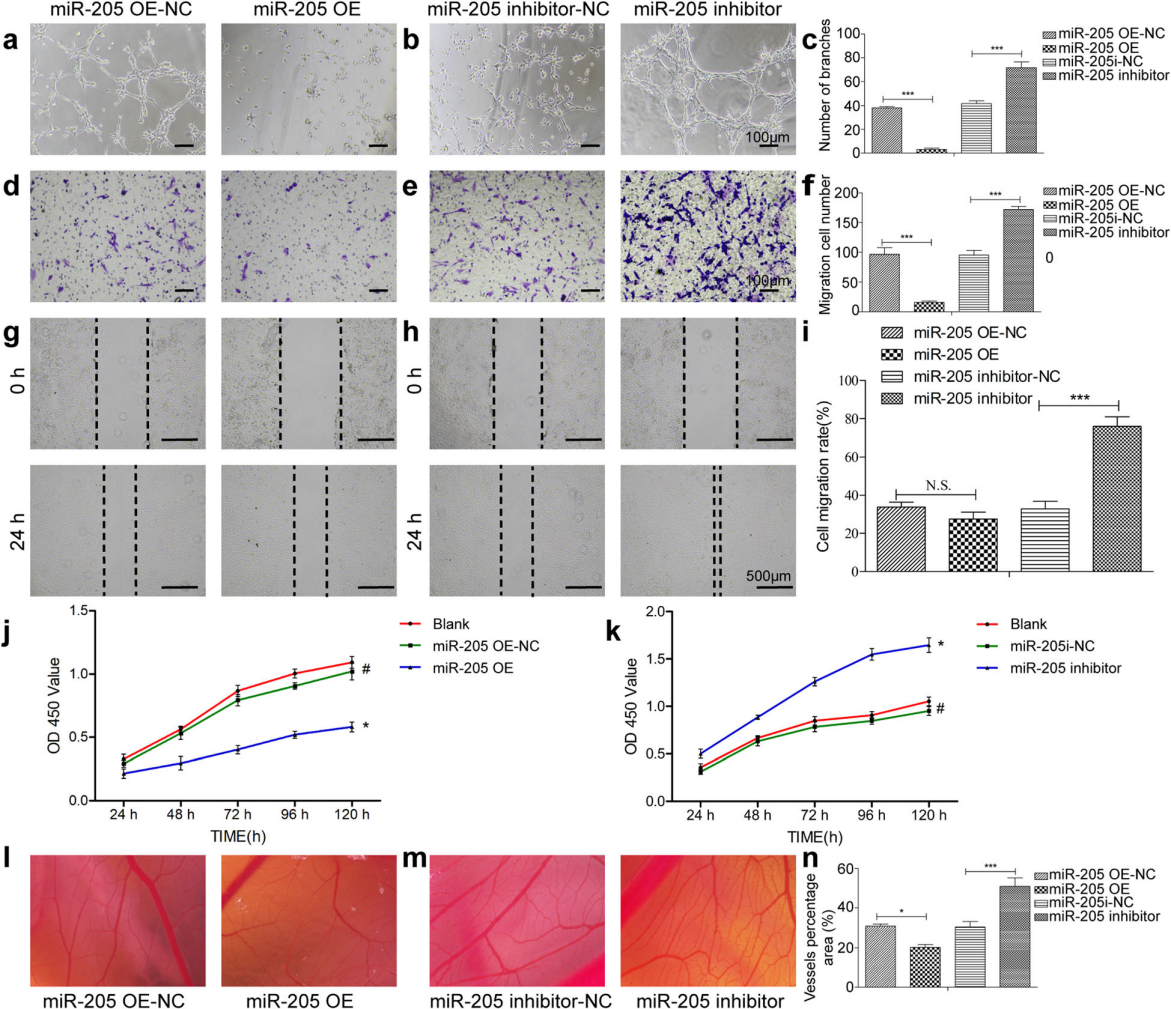
MiR-205 regulates ECFC angiogenesis in vitro and ex vivo. **a**–**c** The results of a Matrigel tube formation assay were assessed 6 h after plating treated ECFCs. **d**–**i** Transwell (**d**–**f**) and wound healing (**g**–**i**) assays confirmed the reduced migration of ECFCs overexpressing miR-205, whereas ECFCs in which miR-205 had been knocked down exhibited enhanced migration. **j**, **k** CCK-8 assay results revealed that miR-205 overexpression significantly reduced the viability of ECFCs (**j**), whereas miR-205 inhibition had the opposite effect (**k**). **l**–**n** CAM assays revealed that miR-205 overexpression significantly decreased vessel density (**l**), while miR-205 inhibition had the opposite effect (**m**). Data are means ± S.D (N.S, no significance; ^#^*P* > 0.05, *P < 0.05, **P < 0.01, and ***P < 0.001; *n* = 3 independent experiments)

Given the close relationship between angiogenesis and osteogenesis, we next isolated ECFCs from canine peripheral blood and identified these cells based on their morphology (Fig. S2A), surface markers (Fig. S2B) and Dil-ac-LDL uptake and FITC-UEA-1 binding assay (Fig. S2C). Then, we explored the role of miR-205 in DO angiogenesis by knocking down or overexpressing this miRNA in ECFCs using appropriate lentiviral vectors which achieved ≥ 80% transduction efficiency as confirmed via fluorescent microscopy. When miR-205 was overexpressed in these ECFCs, they exhibited significantly impaired branch formation in a tube formation assay relative to cells transduced with a control eGFP lentivirus (Fig. 2a), whereas silencing miR-205 markedly enhanced tube formation (Fig. 2b). These data suggested that miR-205 functions to suppress the angiogenic activity of canine ECFCs and that inhibiting miR-205 is thus a viable approach to promoting angiogenesis (Fig. 2c). ECFC migration is a key step in the process of angiogenesis. We found that miR-205 overexpression was associated with impaired ECFC migration in Transwell (Fig. 2d–f) and wound healing (Fig. 2g–i) assays, whereas miR-205 inhibition had the opposite effect. Proliferation is also a key determinant of angiogenesis, and miR-205 overexpression markedly suppressed ECFC cell proliferation in a CCK-8 assay (Fig. 2j), whereas miR-205 inhibition restored the survival and proliferation of these cells (Fig. 2k). To further detect the ex vivo angiogenic ability of these cells, we injected ECFCs that were stably overexpressing miR-205 or its inhibitor into the chorioallantoic membrane (CAM) of chicken eggs (Fig. 2l–n). Chick embryos injected with cells overexpressing miR-205 exhibited a decrease in new vessel density (Fig. 2l). In contrast, when miR-205 expression was inhibited, an increase in new vessel density was observed (Fig. 2m). These findings revealed that elevated miR-205 expression in ECFCs was sufficient to impair vascular stability and angiogenesis, with the knockdown of miR-205 being sufficient to promote angiogenesis in vitro.

### MiR-205 directly suppresses the expression of NOTCH2 and influences angiogenesis

**Fig. 3:**
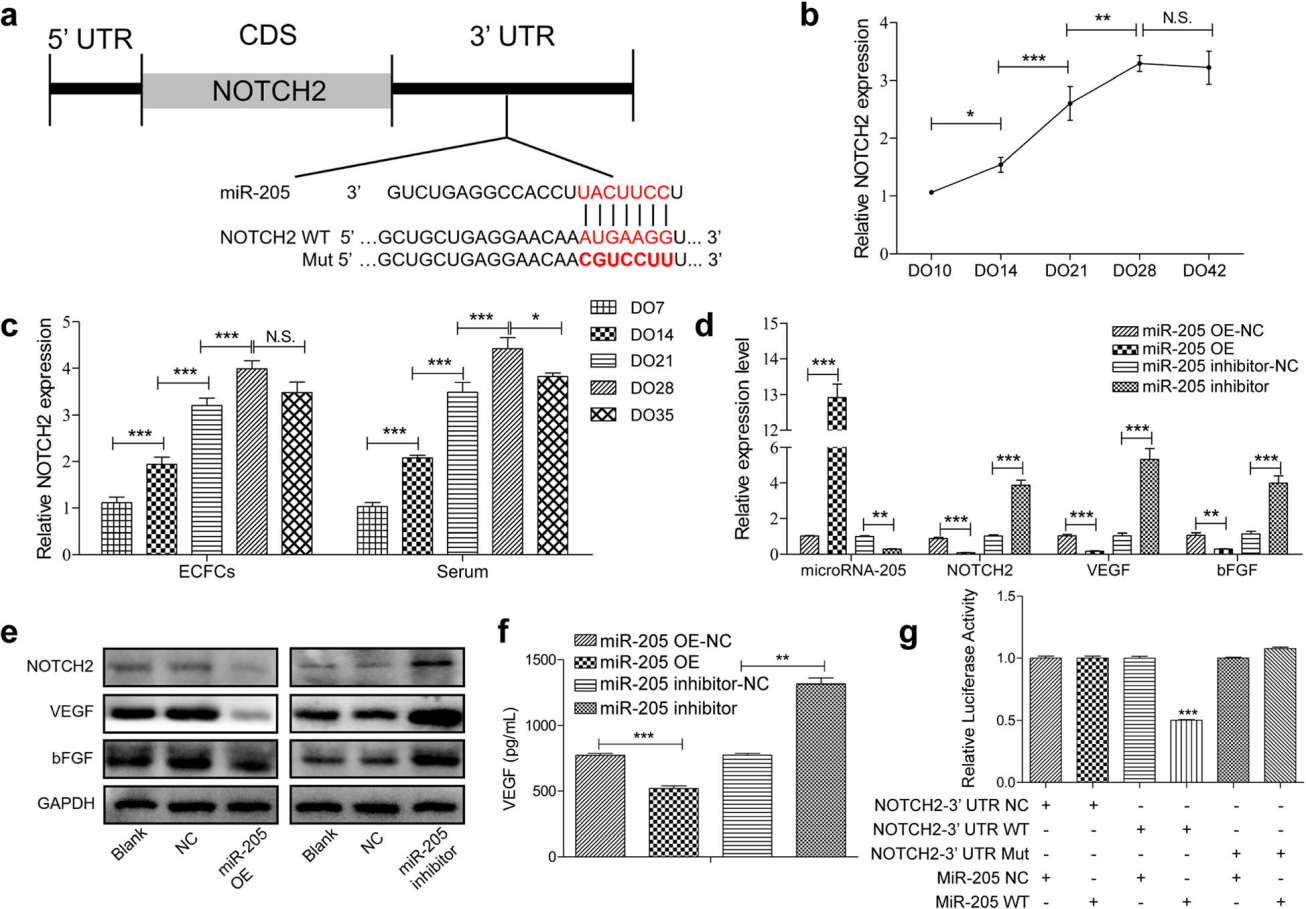
MiR-205 suppresses the expression of NOTCH2 and controls angiogenesis. **a** Sequence alignment of mature miR-205 and the canine NOTCH2 3′UTR. **b** PCR analysis of NOTCH2 mRNA expression levels during the DO process. **c** NOTCH2 expression in ECFCs and serum samples from the peripheral blood. **d**, **e** RNA and protein levels of miR-205, NOTCH2, and angiogenic cytokines were detected by PCR (**d**) and Western blotting (**e**). **f** VEGF secretion from transfected ECFCs was measured via ELISA. **g** Dual-luciferase reporter assays were used to measure relative luciferase activity for wild-type (WT) or mutant (MUT) NOTCH2 reported following miR-205 co-transfection. Data are means ± S.D (N.S, no significance; *P < 0.05, **P < 0.01 and ***P < 0.001; *n* = 3 independent experiments)

To more fully understand the molecular mechanism responsible for the angiogenic function of miR-205, we next queried the TargetScan database (http://www.targetscan.org/vert_72/) to identify putative miR-205 target genes. Included on the list of these target genes was NOTCH2, which was noteworthy given that it is thought to be a unique role in bone angiogenesis [30]. Clear sequence complementarity was observed between miR-205 and the 3′-untranslated region (UTR) of cfa-NOTCH2 (Fig. 3a). When we explored the expression of NOTCH2 in canine distraction callus, we found that it was gradually increased during the distraction and consolidation phases of this process (Fig. 3b). We similarly found NOTCH2 to be upregulated in serum samples and ECFCs isolated from canine peripheral blood during the DO process (Fig. 3c). NOTCH2 and angiogenic cytokine mRNA levels were also negatively correlated with those of miR-205 in ECFC overexpression and knockdown experiments (Fig. 3d), and comparable trends were observed in protein levels (Fig. 3e). An ELISA approach was then used to explore the impact of the miR-205 on VEGF secretion in the context of ECFC angiogenesis, revealing significant increases in levels of pro-angiogenic VEGF in supernatants from ECFCs in which miR-205 had been knocked down, and overexpress miR-205 decrease VEGF secretion (Fig. 3f). The direct repression of NOTCH2 by miR-205 was explored using the dual-luciferase reporter assay, and it was found that miR-205 is able to repress luciferase expression when the construct contained the NOTCH2 3′ untranslated region fused downstream of the luciferase gene (Fig. 3g, the wild-type group). Such repression could be reversed by mutating the predicted miR-205-binding site (Fig. 3g, the mutant group). These results thus identified NOTCH2 as a direct miR-205 target gene in ECFCs and suggested that downregulating miR-205 may increase NOTCH2 expression in DO callus tissues, potentially thereby promoting angiogenesis in the process of MDO.

### MiR-205 controls ECFC function by inhibiting the expression of the transcription factor NOTCH2

**Fig. 4:**
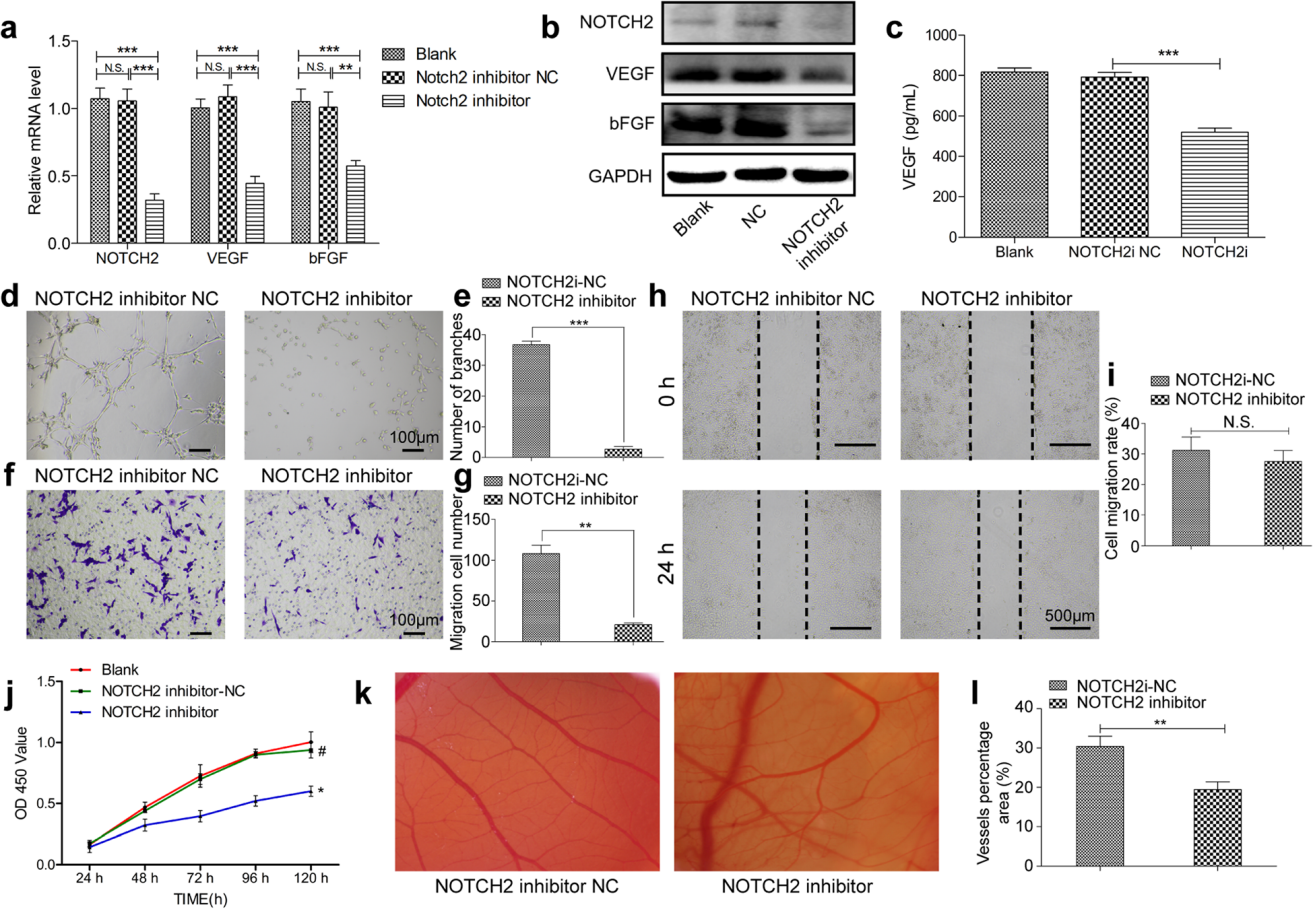
NOTCH2 plays a vital role in ECFC angiogenesis. PCR (**a**) and WB (**b**) analysis of the RNA and protein levels of NOTCH2, VEGF, and bFGF in ECFCs after NC and NOTCH2 inhibitor transfection. **c** ELISA analysis of VEGF secretion from ECFCs after transfection. Tube formation (**d**, **e**), Transwell (**f**, **g**), wound healing (**h**, **i**), and proliferation (**j**) assays revealed that inhibiting NOTCH2 impairs angiogenesis. **k**, **l** CAM angiogenesis assays revealed that when injected with ECFCs in which NOTCH2 was inhibiting, new chick embryo vessel density decreased. Data are means ± S.D. (N.S, no significance; ^#^*P* > 0.05, *P < 0.05, **P < 0.01, and ***P < 0.001; *n* = 3 independent experiments)

**Fig. 5:**
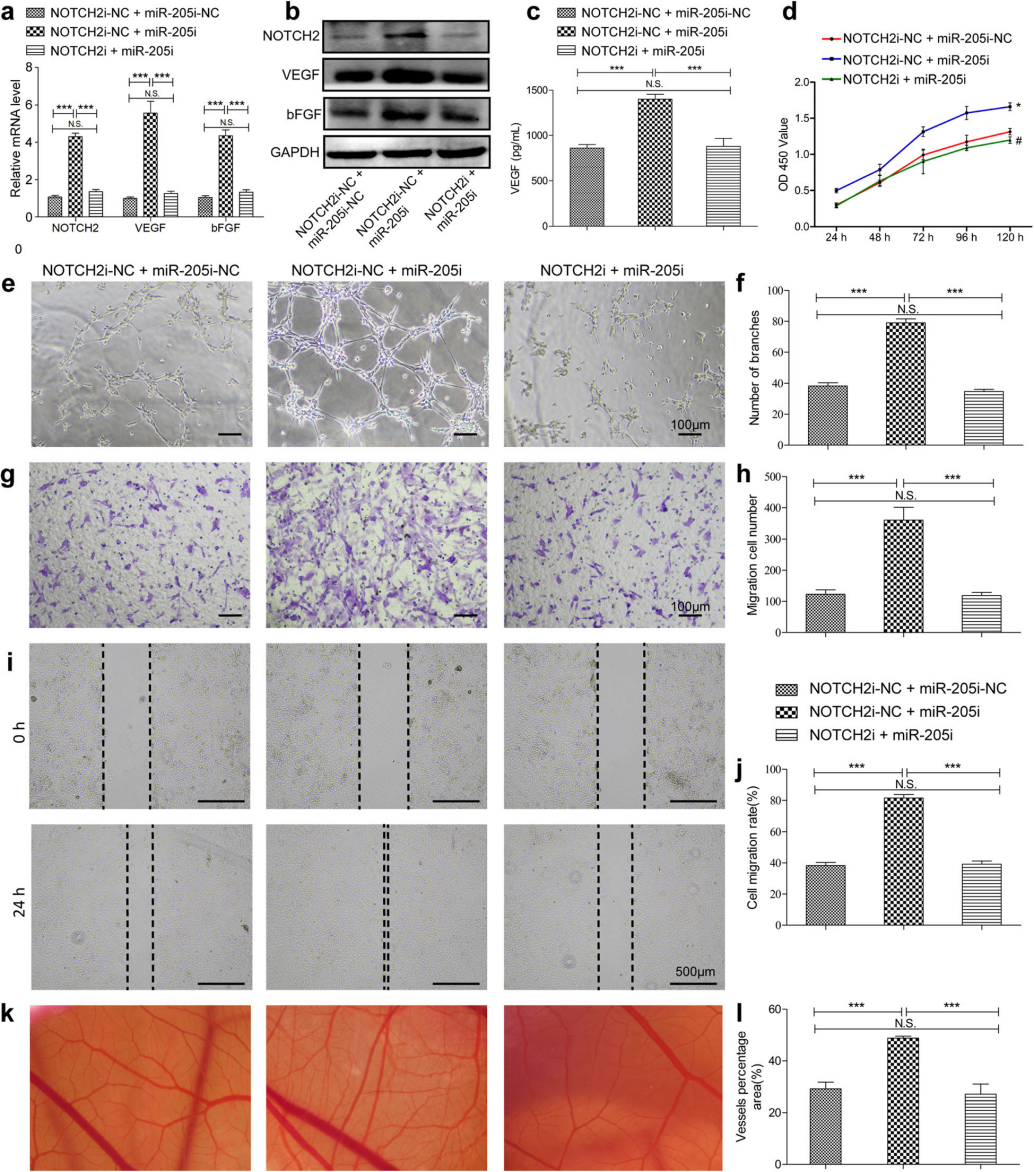
MiR-205 regulates ECFC function via inhibiting NOTCH2. PCR (**a**) and WB (**b**) analyses of the RNA and protein levels of NOTCH2, VEGF, and bFGF in ECFCs after transfection with dual-NC, NOTCH2i-NC + miR-205i, or both inhibitors. **c** VEGF secretion from ECFCs after transfection was measured via ELISA. The results of proliferation (**d**), tube formation (**e**, **f**), Transwell (**g**, **h**), and wound healing (**i**, **j**) assays revealed that miR-205 was able to reverse the angiogenic activity that was impaired by NOTCH2 inhibitor. **k**, **l** Representative photographs of capillaries in CAM samples. Data are means ± S.D. (N.S, no significance; ^#^*P* > 0.05, *P < 0.05, **P < 0.01, and ***P < 0.001; *n* = 3 independent experiments)

In order to firmly establish whether the ability of miR-205 to modulate ECFC angiogenesis was linked to suppress NOTCH2 expression, we next evaluated the functional importance of NOTCH2 in ECFCs. Knocking down NOTCH2 markedly suppressed angiogenic cytokines mRNA and protein levels in ECFCs (Fig. 4a, b), and secreted VEGF levels were similarly suppressed as determined via ELISA (Fig. 4c). Such NOTCH2 knockdown also significantly impaired the tube formation activity (Fig. 4d, e) of these ECFCs, while also suppressing their migration in Transwell (Fig. 4f, g), wound healing assays (Fig. 4h, i), and proliferation ability (Fig. 4j). CAM assay also showed the poor vessel formation ability by inhibited NOTCH2(Fig. 4k, l). NOTCH2 thus plays a key role in facilitating ECFC angiogenesis. Given that we had shown miR-205 to directly suppress NOTCH2 expression, we next treated ECFCs with combinations of lentiviral vectors targeting miR-205 and NOTCH2 in order to more clearly define the regulatory relationship between these molecules. As expected, NOTCH2 and angiogenic gene expression levels were increased in cells transduced with a miR-205 inhibitor construct, whereas simultaneous NOTCH2 knockdown reversed these changes (Fig. 5a, b). ELISA results similarly confirmed that miR-205 inhibition markedly enhanced VEGF secretion, while co-transduction with a NOTCH2 inhibitor lentivirus ablated this increase (Fig. 5c). Suppressing NOTCH2 expression in ECFCs transduced with a miR-205 inhibitor construct similarly suppressed the proliferation (Fig. 5d), tube formation (Fig. 5e, f), Transwell (Fig. 5g, h), wound healing (Fig. 5i, j), and ex vivo vessel formation ability (Fig. 5k, l) of these cells, suggesting that miR-205 regulates angiogenesis by directly inhibiting NOTCH2 expression.

### Knockdown of miR-205 accelerates DO-related angiogenesis and osteogenesis in vivo

**Fig. 6:**
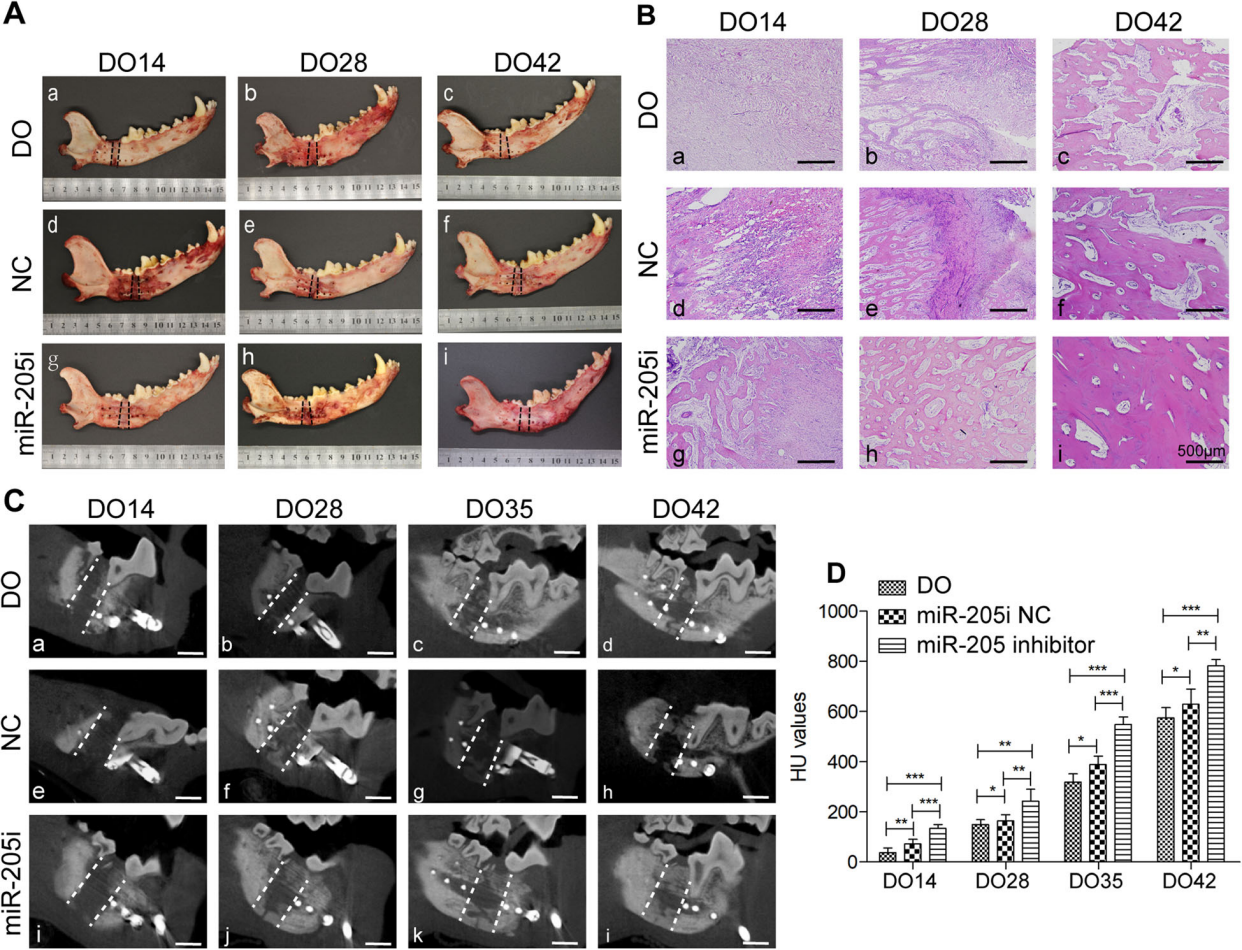
Inhibiting miR-205 accelerates osteogenesis and remodeling during DO. **A** Gross observational result. **B** Histological analyses revealed that inhibiting miR-205 was able to accelerate new bone formation. **C** CBCT results from control, negative control, and miR-205 inhibitor groups following 0, 2, 3, and 4 weeks of consolidation. Few calluses were observed in the distraction gap at the end of the distraction phase (DO14) in these groups. The amount of mineralized callus increased over time, with more callus being evident in the miR-205 inhibited group. The distraction gap was fully bridged at the end of the consolidation phase (bar = 10 mm). **D** Quantitative analyses of CBCT results revealed that the Hounsfield unit (HU) values were significantly increased during DO and were higher in the miR-205 inhibition group relative to the other groups

To explore the functional role of miR-205 during DO in vivo, we locally injected 1 × 10^7^ autologous ECFCs in which this miRNA had been knocked down into the distraction gap at the start of distraction period. Gross observations revealed clear differences in local bone formation in the three different treatment groups, such that the most abundant bone formation in the distraction gap was evident in the anti-miR-205 treatment group, with other groups exhibiting similar but less continuous bone development (Fig. 6A). At the end of distraction (DO14), subsequent H&E staining analyses of the regenerated tissue indicated that the distraction gaps consisted of various amounts of fibrous tissues (Fig. 6B, a, d), while newly formed bone trabecula was observed in miR-205 inhibitor group (Fig. 6B, g). The callus tissue obtained on DO28 in the DO and NC groups were primarily composed of fibrous and new bone trabecula tissues (Fig. 6B, b, e), while the distraction gap samples from the miR-205 inhibition group were bridged by newly formed lamellar bone and few fibrous tissues (Fig. 6B, h). At 4 weeks post-injection (DO42), histological analyses revealed extensive and robust regeneration in the anti-miR-205-modified ECFC treatment group (Fig. 6B, i), whereas calluses in the other groups still exhibited some fibrous or immature callus tissue in the distraction area (Fig. 6B, c, f). When bone remodeling was assessed via CBCT scans in these dogs, bone density (HU values) in the canine mandibular distraction gap was gradually increased, and higher bone density was observed in the miR-205 inhibitor group (Fig. 6C, D). Minimal callus was observed in the distraction gaps immediately after the distraction phase in both the DO and NC groups (Fig. 6C, a, e). After consolidation for 2–3 weeks, new calluses formed from the proximal and distal osteotomy in the NC and miR-205 inhibition groups. More calluses were observed in the anti-miR-205 group relative to the DO and NC groups (Fig. 6C, j, k). By DO42, the distraction gap was fully bridged in animals injected with ECFCs in which miR-205 had been knocked down (Fig. 6C, i), whereas defect gaps were still evident at this same time point in other groups (Fig. 6C, d, h).

## Discussion

While clinically efficacious, DO is limited in clinical settings by its extended duration and the high attendant risks of complications. During the regeneration phase, osteogenesis begins at the sites of vascular sinus formation [31], and there is strong evidence that vasculogenesis and osteogenesis are tightly coupled to one another [32]. Indeed, growth factor-mediated enhancement of angiogenesis or vasculogenesis can accelerate new bone development during distraction [33, 34], whereas impairing angiogenesis can result in bone non-union [35, 36]. By more fully exploring the mechanistic basis for angiogenesis during DO, it may be possible to shorten the consolidation phase of this process and to thereby improve therapeutic outcomes associated with this procedure. Angiogenesis is closely associated with endothelial progenitor cell (EPC) activity in the context of DO. For example, Cetrulo et al. [29] found that EPCs were able to home to ischemic gaps during the distraction phase and to remain there during the consolidation phase in a rat MDO model, while Lee et al. [9, 37] determined that DO promoted the mobilization of bone marrow EPCs into the peripheral blood, whereupon they were able to home to the distraction gap and to promote angiogenesis and bone regeneration in humans and animals. Observed increases in EPC mobilization, together with higher plasma VEGF and SDF-1 levels [33], are consistent with our experimental findings. Overall, these data suggest that increased EPCs homing can augment angiogenesis and thereby promote bone regeneration in the context of DO. While EPCs have been thoroughly studied in this context, their nomenclature in the literature remains inconsistent. ECFCs are generally regarded as the EPCs that are best able to directly facilitate vascular repair at sites of tissue regeneration [11, 38, 39]. There is also robust evidence that miRNA-modified stem cells can drive angiogenesis [40]. ECFCs are thus important mediators of angiogenesis and modulating miRNA expression profiles within these cells has the potential to shorten the DO consolidation phase by altering communication between these endothelial progenitors and nearby osteogenic cells.

Recent studies have explored miRNA expression dynamics and functional roles in the context of a rat limb DO model system [26, 27]. However, the mandible exhibits distinct developmental origins and regenerative characteristics such that it may not be possible to directly extrapolate these findings to a mandibular setting [41]. The mandible is a flat membranous bone that is predominantly cortical and highly dependent on a Haversian vascular network, in contrast to the tubular and primarily trabecular structures of long bones in the limbs which are primarily supplied with blood through a medullary system [41]. Beagle dogs were selected to establish an MDO model in the present study as they are physiologically similar to humans with respect to their size and bone biology [42]. Herein, we found that miR-205 was among the most significantly downregulated miRNAs in the context of MDO. The overexpression of this miRNA was sufficient to suppress ECFC angiogenesis as evidenced by wound healing, migration, and tube formation assays in vitro*.* Bioinformatics analyses led us to identify NOTCH2 as a miR-205 target gene, and we detected significantly higher levels of NOTCH2 expression in distraction callus tissue, serum, and ECFCs at matched time points during DO. We further confirmed that inhibit miR-205 was able to directly increase NOTCH2 expression in vitro, resulting in the elevated secretion of VEGF and thereby accelerating the MDO mineralization process. While endothelial Notch signaling has been shown to suppress angiogenesis in many physiological contexts [43, 44], it instead stimulates angiogenesis and osteogenesis within the skeletal system [30]. Indeed, endothelial Notch reactivation in aged mice has been shown to enhance mineralized bone formation and to bolster vessel-associated osteoprogenitor numbers [45]. Our present findings suggest that NOTCH2, as a miR-205 target within ECFCs, serves as an essential regulator of ECFC-mediated DO angiogenesis. Given the close interactions between osteocyte and endothelial progenitor cells in the context of osteogenesis, further work will be necessary to fully understand the communication between these cell types in this physiological context.

We additionally examined the regenerative activity of miR-205 inhibition in our canine MDO model system. Due to the reaction of immune system against allogenic cells which usually lead to their elimination [12], we isolated autogenous ECFCs from individual dogs, transfected to knockdown miR-205, and injected directly into the distraction callus. CBCT assays revealed that bone anti-miR-205 injection significantly increased bone density on days 28 and 42 of DO. During the consolidation phase, most regenerated woven bone is replaced by mature lamellar bone, while the distracted bone undergoes reshaping. H&E staining confirmed that on DO42, significant amounts of mature lamellar or trabecular bone had been generated in anti-miR-205-treated dogs, whereas notable quantities of immature woven bone trabeculae and fibers were still evident in the other treatment groups. These findings thus indicated that miR-205 inhibition markedly accelerated the bone healing process.

While DO is associated with many clinical advantages, efforts to shorten the consolidation phase of this procedure in humans have been unsuccessful to date. Accelerating angiogenesis represents a promising means of expediting remodeling and thereby shortening this consolidation process. The administration of recombinant protein of NOTCH2 or anti-miR-205 to the distraction gap may represent an effective means of enhancing angiogenic activity in clinical settings, improving local bone regeneration without any systemic side effects. Future studies of the local callus delivery of miRNAs and other recombinant constructs in other preclinical model systems and in human patients will help to close the preclinical gap in translating these findings closer to the clinic.

## Conclusion

**Fig. 7:**
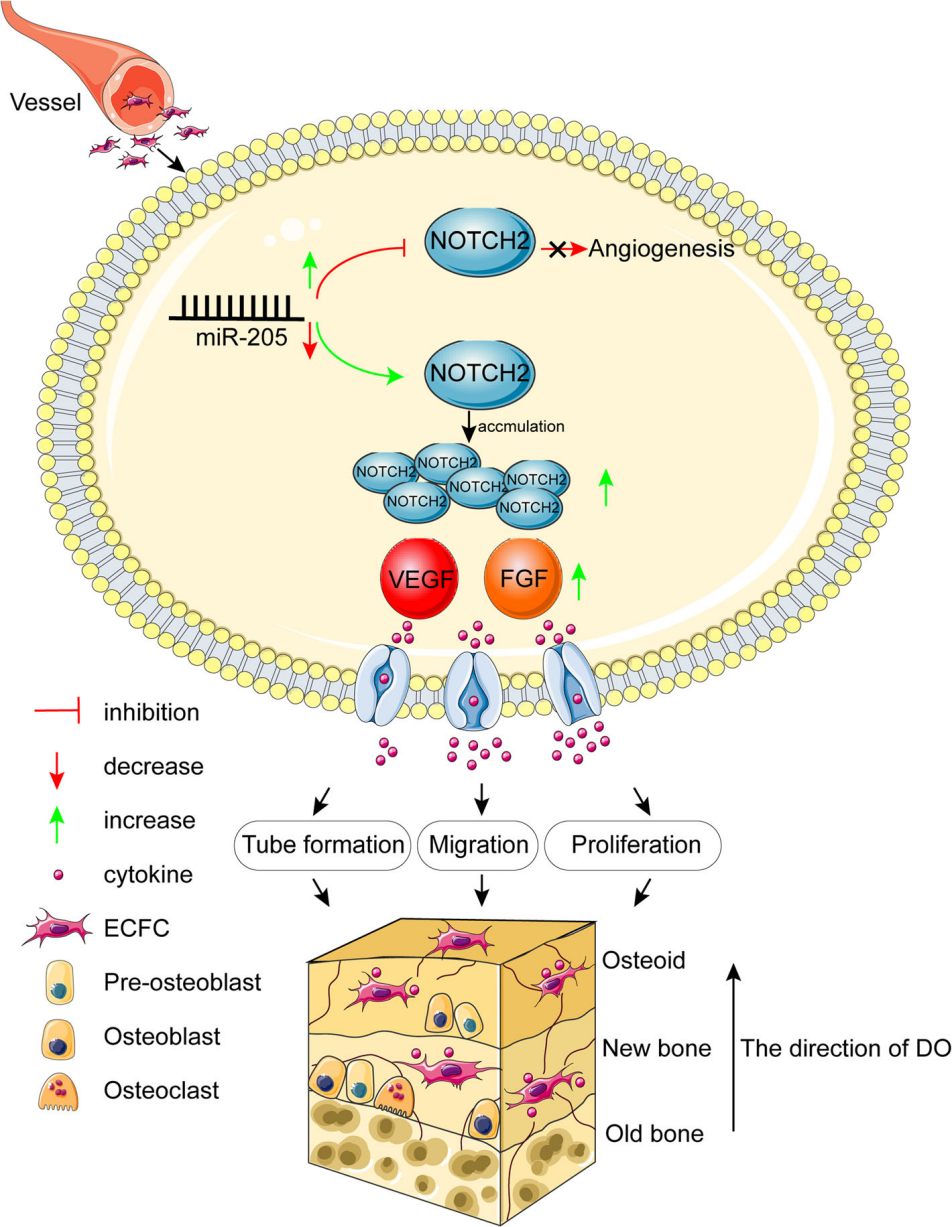
A schematic diagram illustrating the regulation of DO angiogenesis by miR-205. During DO, ECFCs from the peripheral blood express low levels of miR-205. The downregulation of miR-205 results in the increased expression of the transcriptional regulator NOTCH2, further augmenting the secretion of VEGF and bFGF. Increased expression of angiogenic factors by suppressing miR-205, promoting ECFC angiogenesis and thereby accelerating new bone formation and remodeling

In conclusion, we identified miR-205 as a novel regulator of MDO that regulates NOTCH2 expression and thereby shortens consolidation during the process of osteogenesis. Inhibiting miR-205 was sufficient to enhance the proliferative, migratory, and angiogenic activity of ECFCs in vitro through mechanisms linked to NOTCH2 targeting, while the in vivo administration of ECFCs in which miR-205 had been knocked down to the DO callus promoted angiogenesis, accelerated bone regeneration, and local remodeling (Fig. 7). As such, NOTCH2 may represent a promising therapeutic target in the context of DO. Together, these data elucidate key novel signal transduction pathways underlying bone regeneration, highlighting important directions for future clinical research.

## Supplementary Information

**Additional file 1: Figure S1.** Surgical procedure of a canine MDO model.

**Additional file 2: Figure S2.** Characterization of Endothelial colony-forming cells (ECFCs). A The morphology of ECFCs at day 10, 12 and 15 of culture. A Colonies were observed in 10 days (a). By day 12, these cells had been fused in a larger cell monolayer (b) with a cobblestone-like morphology(c). And gradually grew outward at day 15 (d). B Immunophenotypic analyses of ECFCs surface markers (CD31, CD34, CD105, CD45, and CD133) by flow cytometry. C FITC-UEA-1 and DiI-ac-LDL can bind to and taken up by ECFCs, with DAPI used for nuclear staining.

## Data Availability

The datasets used and/or analyzed during the current study are available from the corresponding author on reasonable request.

## Abbreviations

bFGF: Basic fibroblast growth factor
CCK-8: Cell counting kit-8
CBCT: Cone-beam CT
CAM: Chick chorioallantois membranes
DO: Distraction osteogenesis
EPCs: Endothelial progenitor cells
ECFCs: Endothelial colony-forming cells
HU: Hounsfield unit
MNCs: Mononuclear cells
NC: Negative control
OE: Overexpression
PVDF: Polyvinylidene fluoride
RIPA: Radio immunoprecipitation assay
SDS: Sodium dodecyl sulfate
VEGF: Vascular endothelial growth factor
