# Supplementary figures and images for: MicroRNA-205 mediates endothelial progenitor functions in distraction osteogenesis by targeting the transcription regulator NOTCH2
Source: Stem Cell Res Ther. 2021 Feb 3;12:101. doi: 10.1186/s13287-021-02150-x (PMC7860583; doi:10.1186/s13287-021-02150-x)

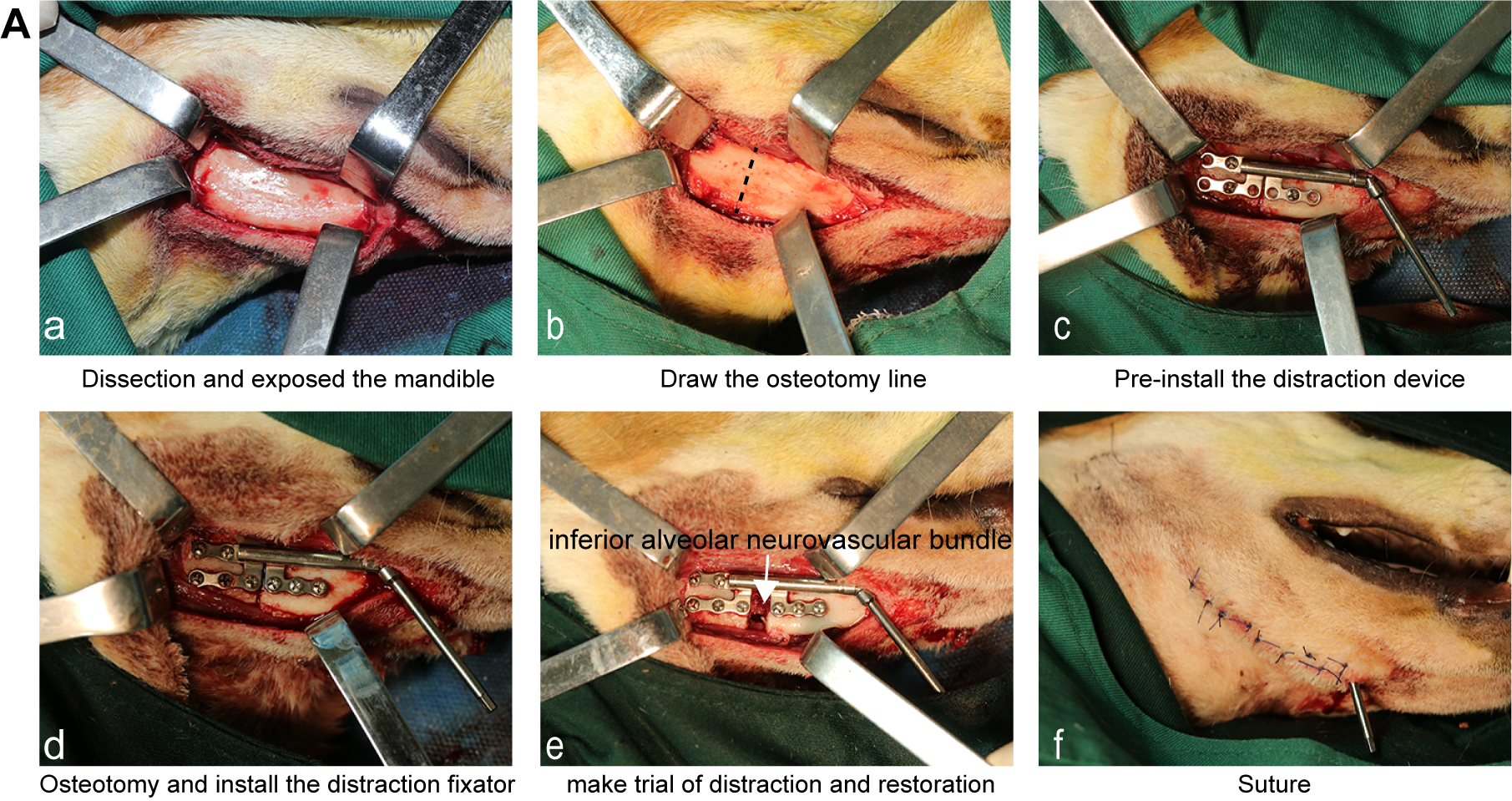

Supplement: Supplementary file 1 — Additional file 1: Figure S1. Surgical procedure of a canine MDO model. [file 13287_2021_2150_MOESM1_ESM.tif]

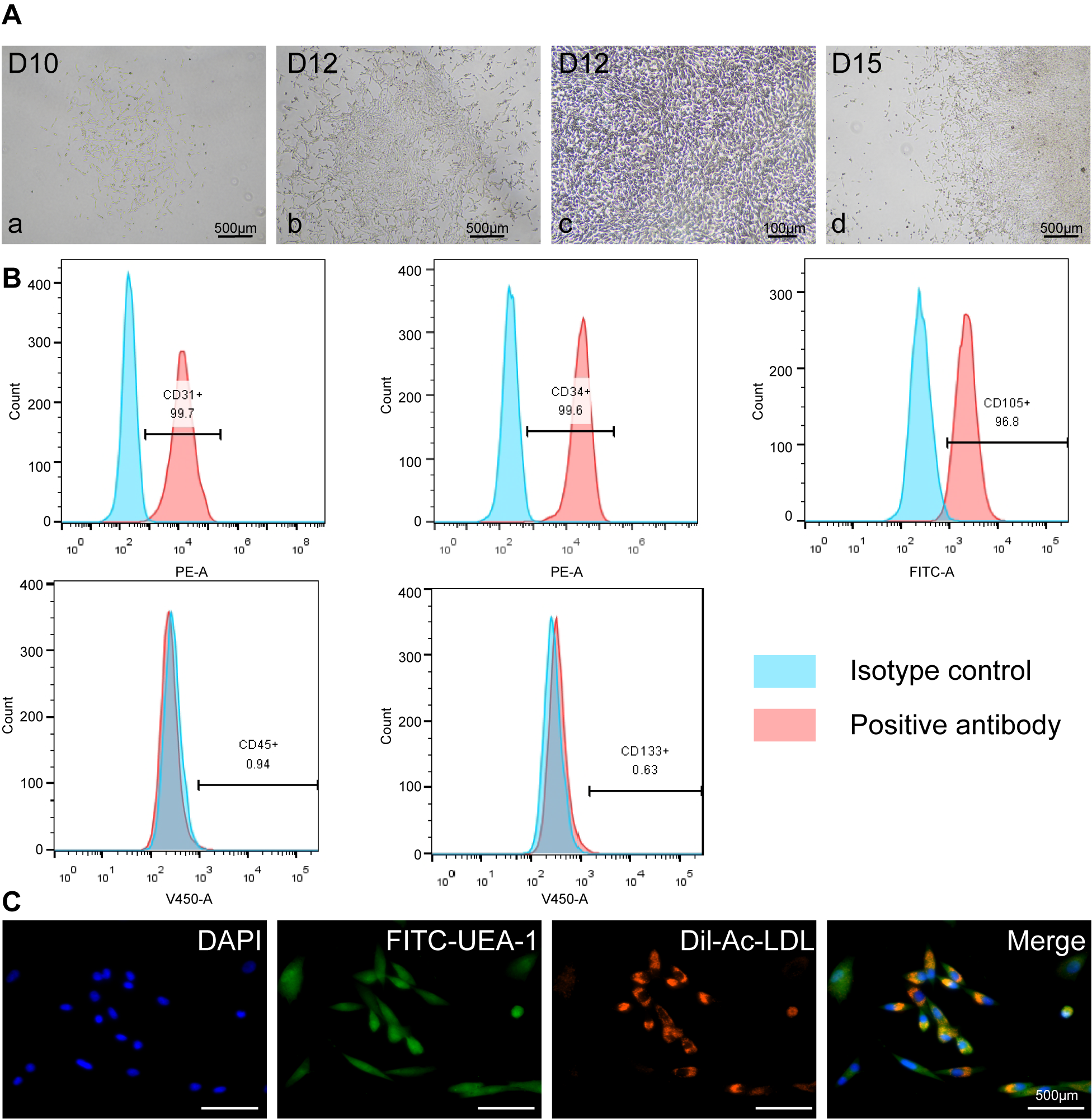

Supplement: Supplementary file 2 — Additional file 2: Figure S2. Characterization of Endothelial colony-forming cells (ECFCs). A The morphology of ECFCs at day 10, 12 and 15 of culture. A Colonies were observed in 10 days (a). By day 12, these cells had been fused in a larger cell monolayer (b) with a cobblestone-like morphology(c). And gradually grew outward at day 15 (d). B Immunophenotypic analyses of ECFCs surface markers (CD31, CD34, CD105, CD45, and CD133) by flow cytometry. C FITC-UEA-1 and DiI-ac-LDL can bind to and taken up by ECFCs, with DAPI used for nuclear staining. [file 13287_2021_2150_MOESM2_ESM.tif]
